# Action of Parasitic Fungi

**Published:** 1882-06

**Authors:** 


### ACTION OF PARASITIC FUNGI.

Mr. Maxime Cornu has recently called attention, in a note read before the Academie des Sciences, to the curious fact that several parasitic fungi cause fallen and decaying leaves to remain green at the spots where they have attacked the plant, when all the rest of the leaf has turned yellow. Microscopical examination shows that the chlorophyll in these spots is in a normal condition, while in the yellowish portion of the leaf it is formed of yellowish globules of altered form. The irritation produced by the parasite preserves the vital activity of the cells from which it derives its nourishment. He considers that this fact throws additional light on the theory that a lichen is an alga stimulated into continued and vigorous growth by the presence of a parasitic fungus.

